# Overexpression of Klotho suppresses liver cancer progression and induces cell apoptosis by negatively regulating wnt/β-catenin signaling pathway

**DOI:** 10.1186/s12957-015-0717-0

**Published:** 2015-10-24

**Authors:** Huidong Sun, Yanchao Gao, Kemei Lu, Guimei Zhao, Xuehua Li, Zhu Li, Hong Chang

**Affiliations:** Department of Hepatobiliary, Surgery Liaocheng People’s Hospital, Liaocheng, 252000 Shandong Province China; Department of Gastroenterology, Liaocheng People’s Hospital, Liaocheng, 252000 Shandong Province China; Department of Hepatobiliary Surgery, Shandong Provincial Hospital Affiliated to Shandong University, Jinan, 250021 Shandong Province China

**Keywords:** Klotho, β-catenin, Apoptosis, Liver cancer

## Abstract

**Background:**

Klotho is a discovered aging suppressor gene, and its overexpression in mice extends the life span of the animal. Recently, Klotho is also identified as a tumor suppressor gene in variety of tumors; however, the potential role and the antitumor mechanism remain unclarified in liver cancers.

**Methods:**

RT-PCR and western blotting analysis were used to detect the expression of Klotho, β-catenin, C-myc, and Cyclin D1. MTT assay was used to detect the survival rates of HepG2 and SMMC-7721 cells. Colony formation assay was used to test the proliferation ability in Klotho transfected cells. FACS was used to detect the cell apoptosis rate in different groups.

**Results:**

The results showed that lower expression of Klotho were found in liver cancer cell lines than the immortalized liver cell L02. Also, MTT assay results found that overexpression or recombinant Klotho administration suppressed the proliferation of liver cancer cells HepG2 and SMMC-7721. Moreover, the colony formation assay results showed that the number of colonies was significantly lower in the cells with transfection with pCMV-Klotho than the controls. Thus, functional analysis demonstrated that Klotho expression inhibited the proliferation of liver cancer cells and Klotho worked as an important antitumor gene in tumor progression. Next, the mechanism was partly clarified that Klotho expression induced cell apoptosis in HepG2 and SMMC-7721 cells, and this phenomenon was mainly involved in the Wnt/β-catenin signaling pathway. The western blotting analysis revealed that overexpression or recombinant administration of Klotho obviously decreased the expression levels of β-catenin, C-myc, and Cyclin D1 in HepG2 cells. Most importantly, the antitumor mechanism for Klotho due to that overexpression of Klotho not only decreased the endogenous β-catenin levels but also inhibited the nuclear translocation of β-catenin to delay the cell cycle progression.

**Conclusions:**

Klotho was a tumor suppressor gene, and overexpression of Klotho suppressed the proliferation of liver cancer cells partly due to negative regulation of Wnt/β-catenin signaling pathway. So, Klotho might be used as a potential target, and the study will contribute to treatment for therapy of liver cancer patients.

## Background

Liver cancer is one of the most lethal cancers in the world, and its mortality ranks the third in cancer deaths [1, 2]. Each year, about 60 million people worldwide die of liver cancer, and this has a growing trend. Primary liver cancers mainly include two types: hepatocellular carcinoma (HCC) and intrahepatic bile duct cancer [3]. In the developing countries, hepatitis B and hepatitis C are the risk factors responsible for most cases of primary liver cancer [4, 5]. Although the risk factors are preventable, the incidence of liver cancer is actually rising in many developing countries. The most common risk factor in the USA is alcohol abuse, followed by obesity and diabetes [6, 7]. The incidence has gotten over 30,000 cases each year in the USA. Thus, it is very important to elucidate the mechanism for the occurrence and development of hepatocellular carcinoma.

Klotho was a new identified aging suppressor gene, which is highly conserved between human and mouse, with 86 % amino acid identity [8]. The gene encoded a membrane protein and defect in Klotho gene expression in the mouse that causes a syndrome of aging, including a short lifespan [9]. Recently, the relationship of Klotho expression and cancer progression has been studied [10–12]; however, the role and mechanism in a variety of cancers remain unclear. Lu et al. had a clinical follow-up study of 189 epithelial ovarian cancer patients, which demonstrated that high expression of secreted Klotho was associated with increased risk of disease progression and death and positively correlated with the expression of IGF-I and IGFBP-3 but not with IGF-II [11]. However, Wang et al. reported that Klotho, as a novel tumor suppressor gene, was epigenetically inactivated and silenced through promoter hypermethylation in gastric cancer, and the promoter methylation of Klotho could be used to predict the prognosis of gastric cancer patients [13]. In human colon cancers, expression of Klotho was downregulated and correlated with tumor invasion and Dukes staging, while overexpression of Klotho inhibited cell proliferation and invasion through inhibition of IGF1R-mediated PI3K/AKT pathway in colon cancer cells [14]. Moreover, Klotho inhibited the capacity of cell migration and invasion in cervical cancer, in vitro restoration of Klotho expression in SiHa cells resulting in a decreased cell motility and invasiveness through upregulation of E-cadherin, downregulation of N-cadherin, and reduced expression of MMP-7 and MMP-9 [15].

However, the relationship and association between the expression of Klotho and primary liver cancers is not clarified till now [16]. In the present study, we explored the effects and possible mechanisms relating to Klotho in human liver cancer cell lines, and the clarification of the association and mechanism would contribute to treatment for therapy of liver cancer patients.

## Methods

### Agent and cell lines

Recombinant human Klotho (rKlotho) is a 65–70 kDa glycoprotein containing 516 amino acid residues. The protein of rKlotho was obtained from Sigma Corporation (cat. SRP3102). The myc-tagged Klotho expression vector (pCMV6-Klotho) and the control vector (pCMV6) were designed and obtained from OriGene corporation (Rockville, MD, USA). The liver cancer cell lines HepG2, SMMC-7721, and BEL-7404 were obtained from American Type Culture Collection (ATCC, Rochville, MD. USA). The normal liver cancer cells L02 was purchased from Junrui Coporation (Shanghai, China). The liver cancer cells were cultured in DMEM medium (GIBCO, Gaithersburg, MD, USA) supplemented with 10 % fetal bovine serum and cultured at 37 °C with 5 % CO_2_ in a humidified atmosphere.

### MTT assay

Cell proliferation is tested by MTT assay. Generally, the human liver cancer cells HepG2 and SMMC-7721 cells were planted into 48-well plates. Then, the cells were cultured for 8 h and transfected with p CMV6-Klotho and p CMV-6 vector for 24, 48, 72, and 96 h. Finally, MTT agent was added into the medium, and the purple crystals were dissolved with DMSO. The liquid was transferred into 96-well plates and read on a microplate reader at a test wavelength of 490 nm and a reference wavelength of 570 nm.

### RT-PCR assay

The RNApure kit (Bioteke, Beijing, China) was used to extract total RNA, and reverse transcriptase (RT) SuperScript III (Invitrogen BV, Carlsbad, CA) was used to transcript the cDNA. The primer sequences were as follows: for glyceraldehyde-3-phosphate dehydrogenase (GAPDH), 5′-GAAGGTGAAGGTCGGAGTC-3′ (sense) and 5′-GAAGATGGTGATGGGATTTC-3′ (antisense) and for Klotho, 5′-ACCTGGTGGCGCACAACC-3′(sense) and 5′-TTGGCAAACCAACCTAGTACA-3′. The Klotho PCR reaction condition is that 94 °C 5 min, followed by 30 cycles of 94 °C for 30 s, 55 °C for 30 s, and 72 °C for 30 s, and finally, elongation is 72 °C for 10 min. The GAPDH reaction condition is similar to Klotho but for the annealing temperature it is 55 °C for 30 s.

### Colony formation assay

The liver cancer cells were transfected with pCMV6-Klotho and pCMV-6 vector for stable cell lines. For colony formation assay, the cells were plated into 6-well plate. After 8 h, the cells transfected with Klotho or control vector were cultured for 14 days, and the medium was refreshed every 3 days. Then, methanol was used to fix the colonies (≥50 cells per colony). They were stained with 1.25 % crystal violet and counted under a light microscope.

### Flow cytometric analysis

Annexin V-FITC/PI dual staining analysis is used for detection of the apoptosis rates in human lung cancer cells according to the kit protocols (Santacruz, USA). Briefly, the liver cancer cells (4 × 105 cells/well) were plated into 6-well plate and transfected with pCMV6-Klotho and pCMV6 vector for 48 h. Then, the cells were washed with ice PBS buffer for three times and resuspended in binding buffer with HEPES–NaOH 10 mM pH 7.4, 25 mM CaCl2, and 144 mM NaCl. At last, the staining dye of Annexin V (0.1 μg/μl) and PI (0.05 μg/μl) were added for incubation in the dark for 30 min on ice, and the cells were subjected to FACS analysis.

### Western blot analysis

The liver cancer cells were plated into 48-well plates and cultured for 8 h for adherence. Then, the cells were transfected with pCMV6-Klotho and p CMV-6 vector for 48 h. The pCMV6 vector was used as the control. The other treatment was that the cells were treated with rKlotho or BSA at the concentration of 250 ng/mL. BSA was used as the negative control. Next, the cells were washed with ice-cold PBS buffer, and cell lysates were prepared for PAGE. The primary antibodies used were included; the anti-beclin 1, anti-LCI, anti-LC-II, anti-C-myc, anti-Cyclin D1, and anti-GAPDH were all purchased from Santa Cruz biotechnology. The primary antibodies of β-tublin (ab6046), lamin B1(ab16048), and Anti-Klotho (ab18131) were obtained from Abcam corporation. The horseradish peroxidase-conjugated goat anti-mouse secondary antibody was purchased from Abgent Corporation.

### Statistical analysis

All of the data was analyzed using SPSS software. All of the experiments were repeated three times in duplicates. Student’s *t* test was used to assess the significance of the data, and all of the data are showed as mean ± SD. *P* < 0.01 means that there is significant difference.

## Results

### Low level of Klotho is detected in liver cancer cell lines

**Fig. 1:**
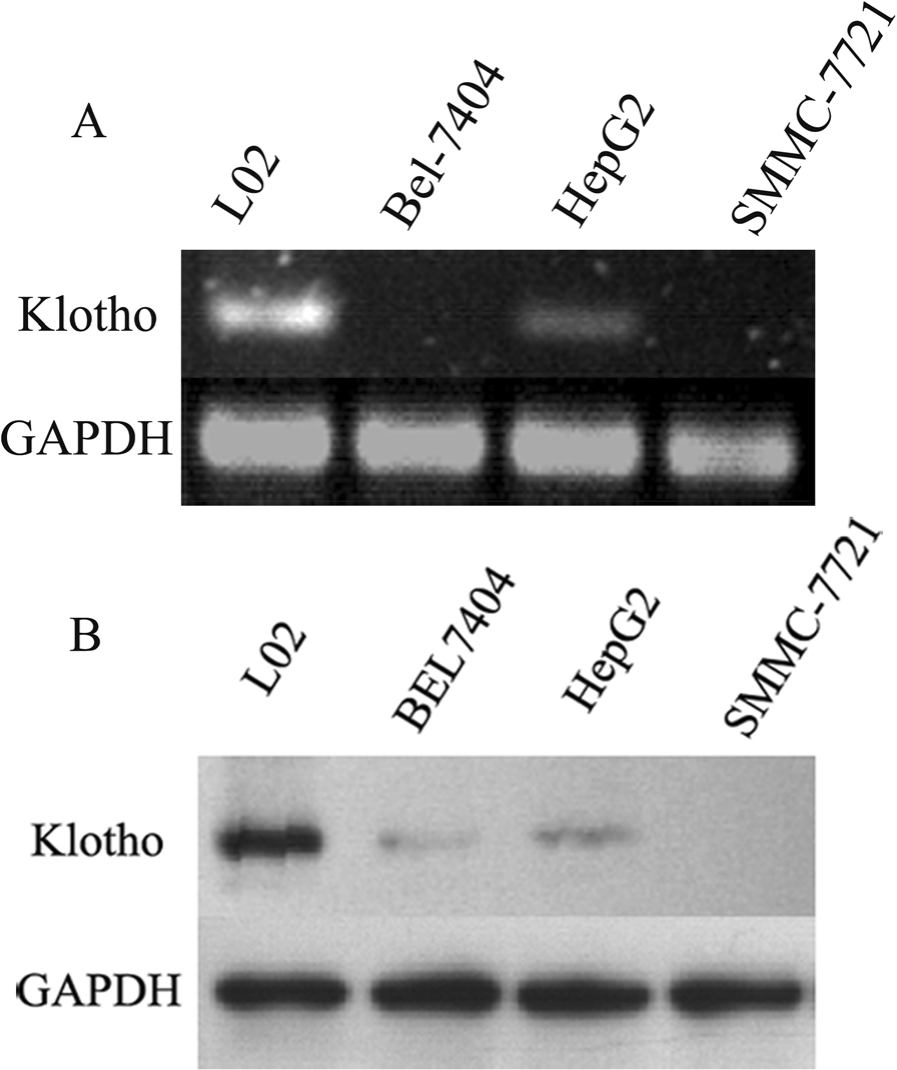
Low level of Klotho is detected in the liver cancer cell lines. The liver cancer cells (5 × 10^5^ cells/well) were plated into 6-well plate. After 8 h, the endogenous Klotho levels in a panel of four liver cancer cell lines were detected with RT-PCR (**a**) and western blotting analysis (**b**). L02 is an immortalized liver cell line, and GAPDH is used as an internal reference

To explore the Klotho expression of Klotho in liver cancer cell lines, we analyzed Klotho levels in a panel of four liver cancer cell lines by RT-PCR and western blotting analysis. Low mRNA levels and protein levels were detected in liver cancer cell lines, such as Bel-7404, HepG2, SMMC-7721 etc., compared with the immortalized liver cell L02 (Fig. 1).

### Overexpression or recombinant Klotho administration suppresses the proliferation of liver cancer cells

**Fig. 2:**
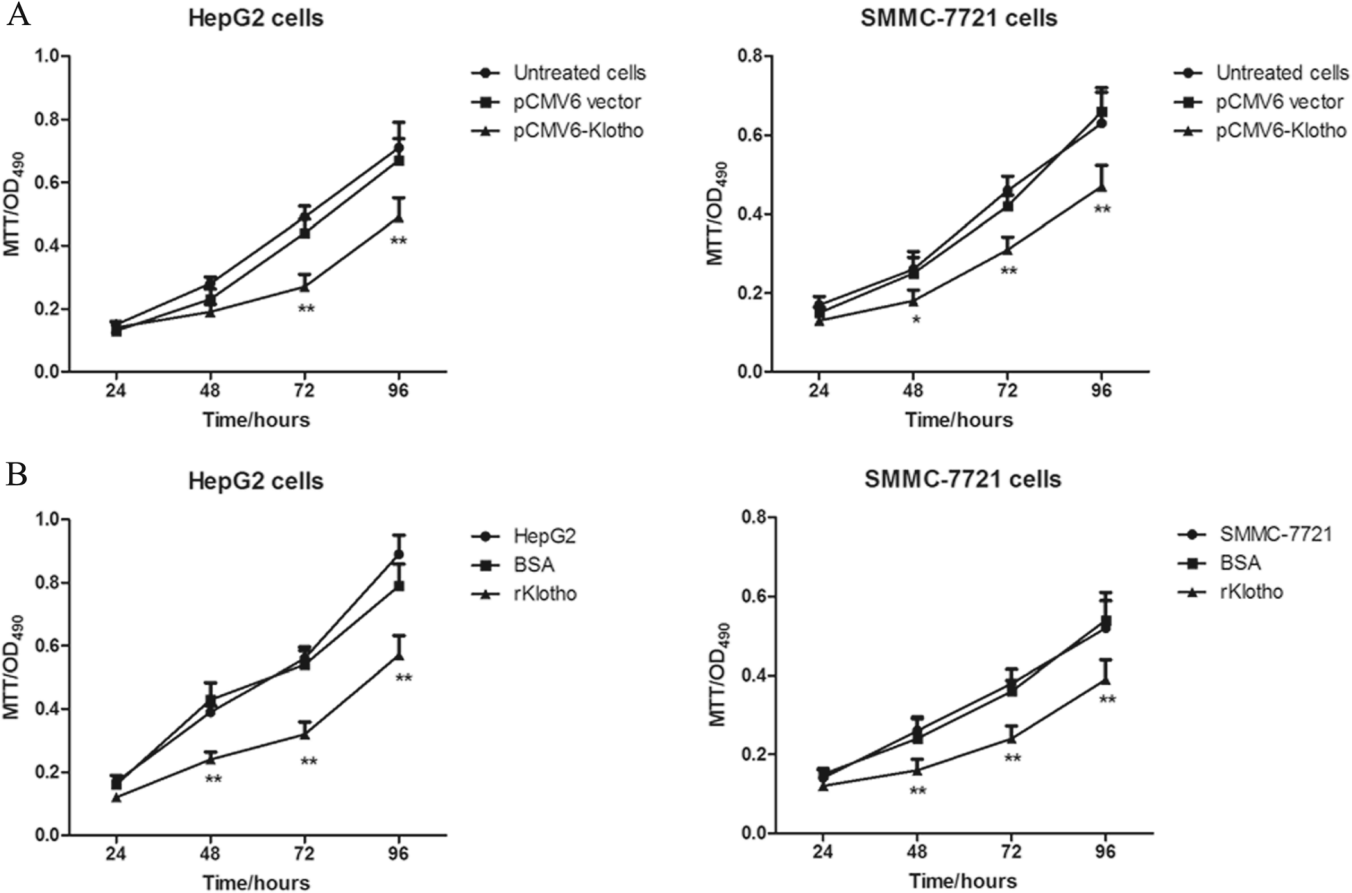
Overexpression or recombinant Klotho administration suppresses the proliferation of liver cancer cells. **a** The liver cancer cells (2 × 10^4^ cells/per well) were plated into 48-well plate. After 8 h, the cells were transfected with p CMV6-Klotho and control vector for 24, 48, 72, and 96 h. The cells transfected by p CMV6 vector were used as the controls. Data are shown as means ± SD. **p < 0.01 compared with control cells. **b** The liver cancer cells HepG2 and SMMC-7721 (1 × 10^3^ cells/per well) were plated into 96-well plate. After 8 h, the cells were treated with recombinated Klotho protein and control protein BSA for 24, 48, 72, and 96 h. Data are shown as means ± SD. **p < 0.01 compared with the cells treated with BSA

We next evaluated the effect of Klotho on cell proliferation of liver cancer cells. HepG2 and SMMC-7721 cells were transfected with expression vector of pCMV6-Klotho for 24, 48, 72, and 96 h. As shown in Fig. 2a, the OD490 values were obviously decreased in the group transfected with p CMV6-Klotho than those of p CMV6 vector, suggesting that the cell proliferation was significantly suppressed as higher expression of Klotho. Additionally, as shown in Fig. 2b, the HepG2 cells and SMMC-7721 cells were treated with recombinant Klotho at the concentration of 300 ng/mL for 24, 48, 72, and 96 h. The results demonstrated that rKlotho administration inhibited cell growth of liver cancer cells HepG2 and SMMC-7721, and higher expression of Klotho was accompanied with lower proliferation of liver cancer cells. All of the data collectively revealed that Klotho expression inhibited the proliferation of liver cancer cells.

### Klotho expression suppresses liver cancer cell growth by colony formation assay

**Fig. 3:**
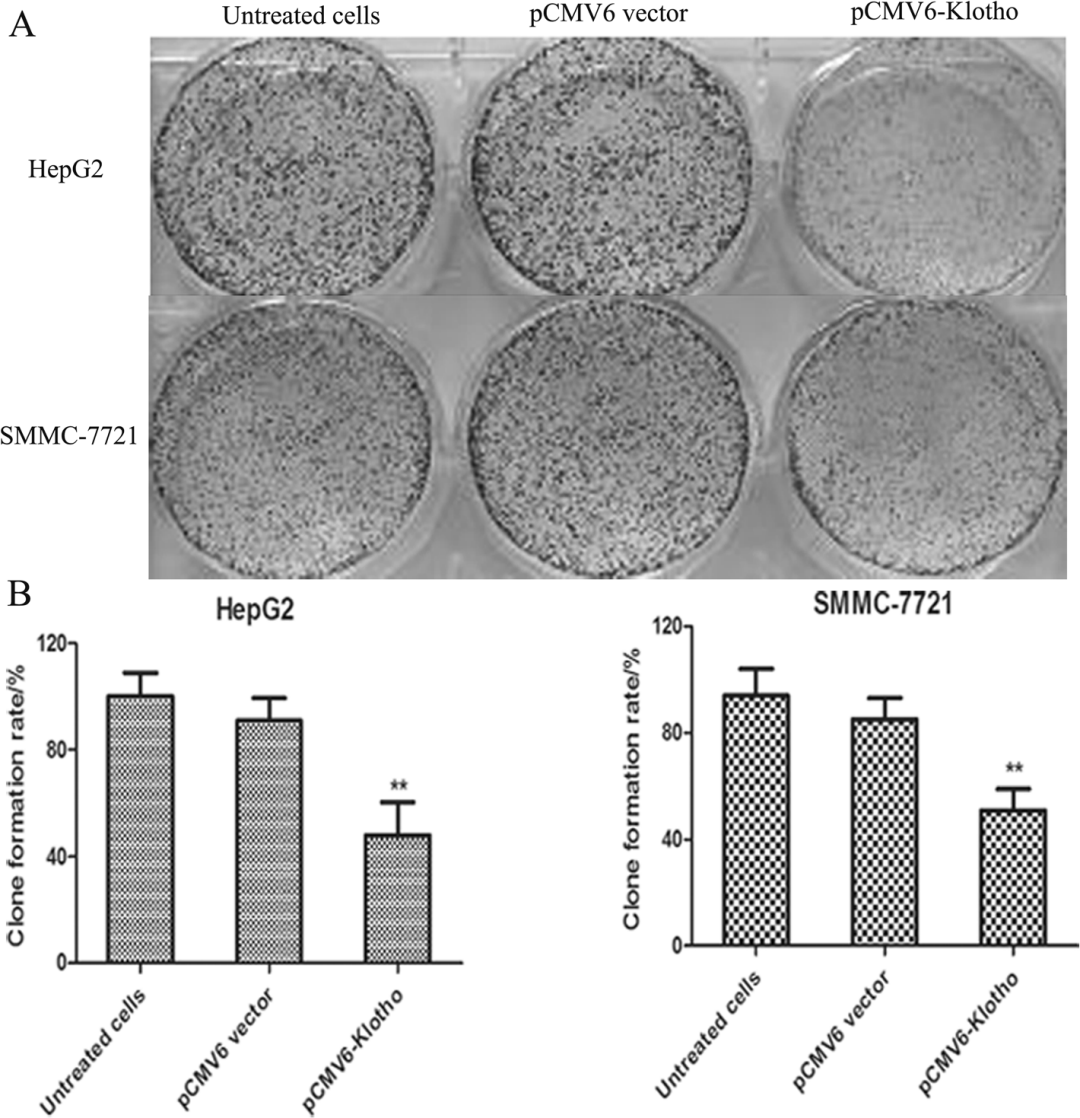
Klotho expression suppresses liver cancer cell growth by colony formation assay. **a** The liver cancer cell line of HepG2 and SMMC-7721 was used for colony formation assay. **b** Histogram of clone formation ratio was shown in pCMV6-Klotho group, control plasmid group, or untreated group. **p < 0.01, compared with the cells transfected with pCMV6-vector

In order to clarify the functional relevance of Klotho in liver cancer progression, colony formation assay is performed in liver cancer cells after Klotho expression. The liver cancer cell line HepG2 and SMMC-7721 were transfected with p CMV6-Klotho and control plasmid (pCMV6 vector), and the cells were cultured for 10 days. As shown in Fig. 3, the number of colonies was significantly lower in the cells with transfection with pCMV-Klotho than the controls. Taken together, Klotho expression inhibits the proliferation of liver cancer cells, and Klotho plays an important antitumor role as a potential tumor suppressor.

### Klotho expression induces cell apoptosis in human liver cancer cells

**Fig. 4:**
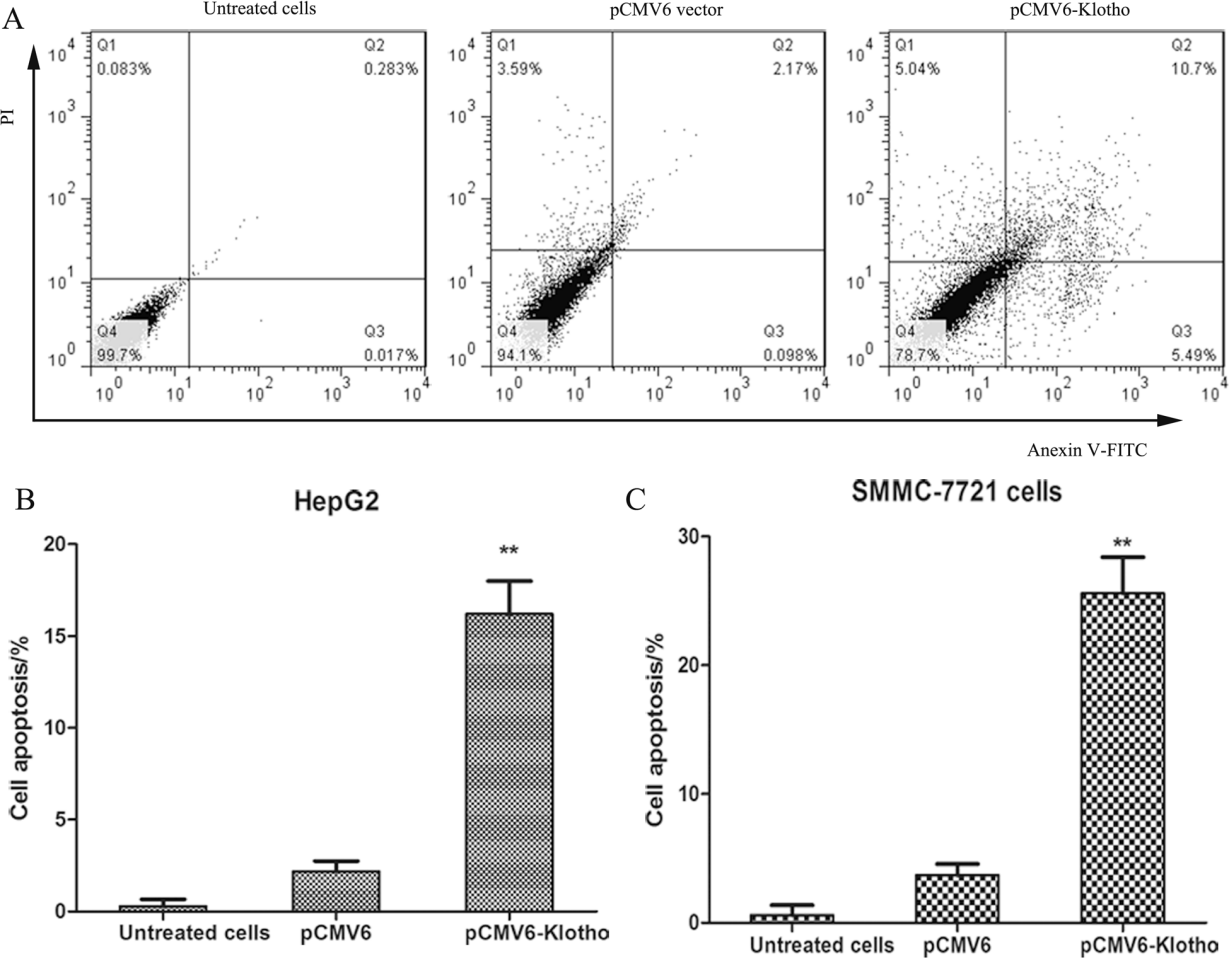
Klotho expression induces cell apoptosis in human liver cancer cells. **a** The HepG2 cells were plated into 6-well plate and were transfected with pCMV6-Klotho and pCMV6 vector for 48 h. Then, the cells were fixed, dispersed, and stained with Annexin V and PI. The cell apoptosis rate was detected by flow cytometry analysis. The histogram of HepG2 (**b**) and SMMC-7721 (**c**) were shown. The cells transfected with pCMV6 vector were used as negative controls. **p < 0.01, compared with negative control

It is proved that overexpression or recombinant Klotho administration could inhibit the proliferation of liver cancer cells. In order to identify whether cell apoptosis is induced in cancer cells with overexpression of Klotho, FACS analysis was used by dural staining with PI and Annexin V-FITC. As shown in Fig. 4, the apoptosis rates in HepG2 cells transfected by p CMV6-Klotho was significantly higher than the control cells (*P* < 0.01), and this was consistent with the situation in SMMC-7721 cells.

### Klotho levels negatively correlate with Wnt/β-catenin signaling pathway in HepG2 cells

**Fig. 5:**
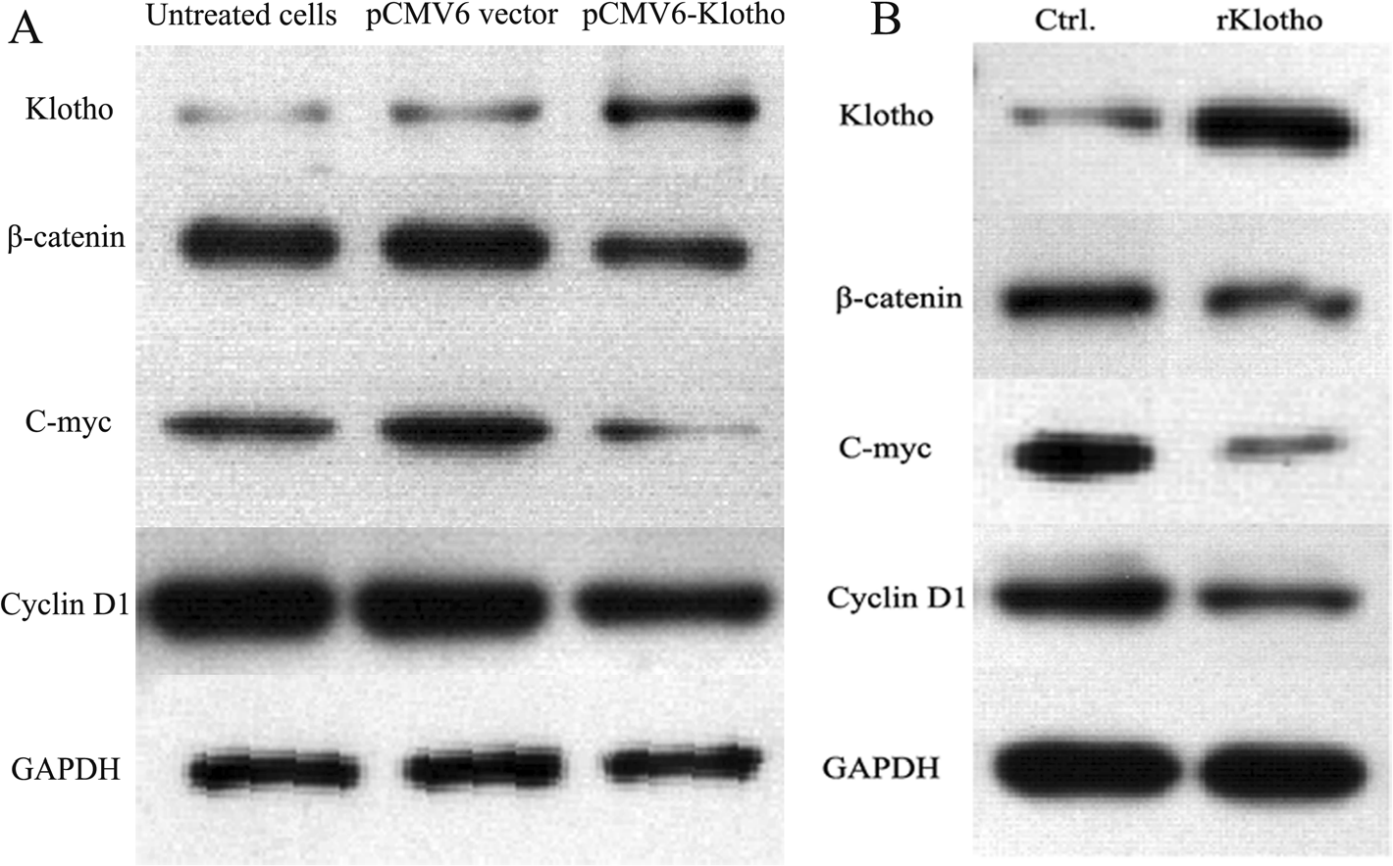
Klotho levels negatively correlate with Wnt/β-catenin signaling pathway in HepG2 cells. **a** The HepG2 cells with the density of 3 × 10^4^ cells/per well were plated in 24-well plates. Eight hours later, pCMV6-Klotho and p CMV6 vector were transfected into HepG2 cells. After 48 h, the expression of Klotho, β-catenin, C-myc, and Cyclin D1 was detected by western blotting. **b** The HepG2 cells with the density of 1 × 10^3^ cells/per well were plated in 24-well plates. Eight hours later, recombinated Klotho and BSA were added into HepG2 cells, respectively. After 48 h, the expression of Klotho, β-catenin, C-myc, and Cyclin D1 was detected by western blotting

It is reported that Wnt/β-catenin signaling pathway is abnormally expressed in liver cancer cells. In order to explore the relationship between the tumor suppressor, Klotho and Wnt/β-catenin signaling pathway, the HepG2 cells were transfected with p CMV6-Klotho and control plasmid p CMV6. The expression levels of Klotho, β-catenin, C-myc, and Cyclin D1 were detected by western blot. As shown in Fig. 5a, the HepG2 cells in p CMV6-Klotho transfected group had higher levels of Klotho, accompanied by lower expression of β-catenin, C-myc, and Cyclin D1. Consistent with this finding, the HepG2 cells were treated with recombinated Klotho for 48 h, and the results showed increased Klotho expression concomitant with decreased levels of β-catenin, C-myc, and Cyclin D1 (Fig. 5b).

### Overexpression of Klotho decreases endogenous β-catenin level and inhibits its nuclear translocation

**Fig. 6:**
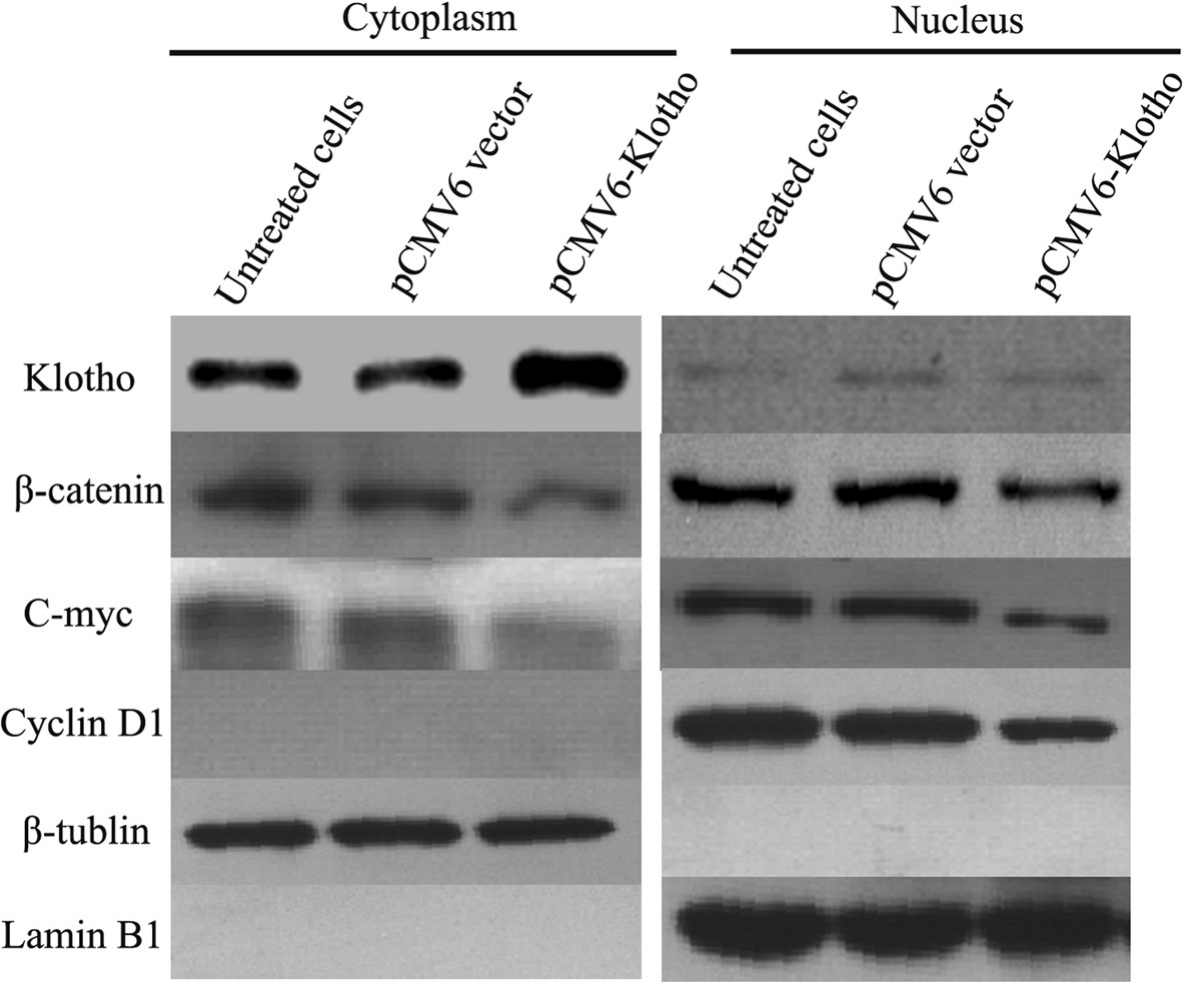
Overexpression of Klotho decreases endogenous β-catenin level and inhibits its nuclear translocation. The liver cancer cells (2 × 10^3^ cells/per well) were plated into 6-well plate, and the cells were transfected with pCMV6-Klotho and the control plasmid after 8 h. Cell lysates were prepared for detecting of β-catenin in cytoplasm and nucleus by western blot analysis after transfection for 48 h. And, the expression levels of cyclin D1 and C-myc were also detected by western blotting analysis. β-tublin was used as an internal reference in cytoplasm, and lamin B1 was used as the internal reference in nucleus

The activation of Wnt/β-catenin signaling pathway is that β-catenin was translocated into the nucleus. Next, we wanted to know whether overexpression of Klotho would affect the translocation of β-catenin. Thus, we detected the distribution of β-catenin in cytoplasm and the nucleus. As shown in Fig. 6, in pCMV-Klotho-transfected HepG2 cells, the expression levels of β-catenin obviously increased in cytoplasm compared with those in control cells. The β-catenin levels in cytoplasm and nucleus were both decreased in p CMV6-Klotho transfected cells, accompanied by lower expression levels of C-myc and Cyclin D1. All of the data revealed that overexpression of Klotho decreased the endogenous expression of β-catenin and inhibited the translocation of β-catenin from cytoplasm into nucleus. Additionally, the transcription of target genes in the downstream of wnt/β-catenin was also downregulated, such as C-myc and Cyclin D1, which was involved in cell cycle progression.

## Discussion

Primary hepatocellular carcinoma is a common malignancy in the world [17]. With the emergence of the problem, such as an aging population and environmental pollution, the incidence of liver cancer arises rapidly and liver cancer has become the second killer in China [18, 19]. It not only gives the pain and psychological burden for patients but also gives serious economic burden to the family and the community [20, 21]. Thus, it has become the focus of medical research and hotspots in China to investigate the molecular mechanisms of the development and progression of liver cancer, which could give some new clues for the prognosis and treatment on hepatocellular carcinoma.

Klotho has been newly identified as an antiaging gene that is involved in the suppression of several aging phenotypes [22–25]. But the potential role and the antitumor mechanism remains unclear in human liver cancers. In the present study, we first explored the expression levels in a panel of three liver cancer cell lines, HepG2, SMMC-7721, and Bel-7404, and the normal liver cell L02. As expected, the expression levels of Klotho in three liver cancer cell lines were obviously decreased than those in L02 cells. The MTT assay proved that overexpression or recombinant Klotho administration in liver cancer cells significantly suppressed the proliferation of cancer cells. This was consistent with the results from the colony formation assay. Moreover, FACS analysis results demonstrated that overexpression of Klotho induced cell apoptosis in HepG2 and SMMC-7721 cells. All of the data revealed that Klotho worked as an important antitumor gene in liver cancer progression although some other paper found that high levels of Klotho promoted cancer progression. One of the probable reasons is that Klotho may have different role in the progression and development of various tumors.

Then, we further explore the mechanism of Klotho involving in the progression of liver cancers. It has reported that overexpression of Klotho inhibits growth and invasion through inhibition of IGF1R-mediated PI3K/AKT pathway in colon cancer cells [14]. In lung cancer cells, Klotho, as a novel secreted Wnt antagonist, could inhibit activation of Wnt /beta-catenin signaling pathway, in a dose-dependent manner [23]. As we know, Wnt/β-catenin signaling pathway is essential for development and tumorigenesis, and this signaling pathway is abnormally activated in the progress of hepatocellular carcinoma. Beta-catenin is an important component in Wnt/β-catenin pathway by being a coactivator of LEF/TCF transcription factors. In pathological conditions, cytoplasmic β-catenin leads to its nuclear accumulation and form the complex with LEF/TCF transcription factors. Then, the LEF/TCF target genes are transactivated, such as C-myc and Cyclin D1, which promote cell cycle progression.

Here, we also detected the association of Klotho expression with Wnt/β-catenin signaling pathway. The western blotting analysis showed that high levels of Klotho were associated with decreased levels of β-catenin. Importantly, the nuclear translocation of β-catenin was also suppressed in HepG2 cells. Additionally, the target genes of the downstream (C-myc and Cyclin D1) were downregulated. All of the data demonstrated that overexpression of Klotho suppressed the proliferation of liver cancer cells and promoted cell apoptosis partly due to negative regulation of Wnt/β-catenin signaling pathway. Thus, Klotho might be used as a potential target, and the study will contribute to treatment for therapy of liver cancer patients.

## Conclusions

Klotho was a tumor suppressor gene and overexpression of Klotho suppressed the proliferation of liver cancer cells partly due to negative regulation of Wnt/β-catenin signalling pathway. So, Klotho might be used as a potential target and the study will contribute to treatment for therapy of liver cancer patients.
